# Physical and Psychological Discomfort Experienced by Hematopoietic Stem-Cell Donors

**DOI:** 10.3390/ijerph17072316

**Published:** 2020-03-30

**Authors:** Miok Kim, Tai-Gyu Kim, Su-Hee Beom

**Affiliations:** 1 College of Nursing, Dankook University, Cheonan 31116, Korea; 2 College of Medicine, Catholic University, Seoul 06591, Korea; kimtg@catholic.ac.kr (T.-G.K.); ulsu777@catholic.ac.kr (S.-H.B.)

**Keywords:** hematopoietic stem cells, peripheral blood stem cell, unrelated donors

## Abstract

This study investigates the types and degrees of physical and psychological discomfort experienced by hematopoietic stem cell donors before, during, and after the donation process in order to provide helpful information for developing education programs that can help donors to cope with their discomforts. One hundred and thirty-one individuals who donated hematopoietic stem cells from 2017 to 2019 were asked to self-report the types and degrees of physical and psychological discomfort they felt in the process, and the results were analyzed using SPSS. All participants donated peripheral blood stem cells; the most commonly reported physical discomfort was myalgia (72.5%), followed by bone pain (62.6%), fatigue (60.3%), and headache (55.0%). Of the donors, 88.5% responded that they experienced psychological discomforts, including fear (44.3%), anxiety (44.3%), stress (39.7%), depression (31.3%), loneliness (31.3%), regret (29.8%), and ambivalence (23.7%). In particular, female donors experienced more discomfort than males in rash (Z = −2.123, *p* = 0.034), fear (Z = −2.851, *p* = 0.004), and anxiety (Z = −1.861, *p* = 0.044). Therefore, it is necessary for healthcare providers and experts to make efforts to educate and help donors to prepare and mitigate their discomfort throughout the donation process, and to strategically manage donors’ well-being by monitoring and evaluating their discomfort levels and providing interventions if necessary.

## 1. Introduction

Hematopoietic stem cell transplantation (HSCT) is the standard care procedure for congenital or acquired disorders of the hematopoietic system, as well as malignant diseases sensitive to anticancer agents, radiation therapy, and immunotherapy. The number of HSCTs has increased with the expansion of indications for transplant, use of new sources, and development of novel conditioning regimes [1]. In South Korea, approximately 2800 patients each year require HSCT due to hematologic diseases, such as leukemia, aplastic anemia, and metabolic disease [2].

For HSCT to work, there must be a match between the human leucocyte antigens (HLAs) of the recipient and donor. There is a 25% chance that siblings will be HLA matched, 5% chance that a parent and child will be HLA matched, and, on average, only about one in twenty thousand chance that two unrelated individuals will be HLA matched [3]. Therefore, having sufficient donors is fundamental in order to meet the large demand of HSCT candidates. According to the 2016 Korean Network for Organ Sharing (KONOS) statistics, approximately 313,000 potential HSC (Hematopoietic Stem Cell) donors have been registered, which is not enough, considering the chance of finding an HLA-matched donor. Moreover, even when a HLA-type matched potential HSC donor is found, the average consent rate for actual donation has been around 58.1% for the past 20 years, showing a decreasing trend in the last three years (2012–2014), from 55.1% to 54.0% and to 51.0% [4].

Personal motives, parent’s objections, health problems, and the inability to contact the appropriate channels have been reported as reasons for not consenting to donate [2]. A large-scale study [5] of potential HSC donors registered in HSC donation-related organizations, including the United States’ National Marrow Donor Program (NMDP), indicated that ambivalence about donation was the most consistent factor associated with opting out of the registry. It seems volunteer donors are often insecure and hope that people other than themselves will donate, possibly because of doubts, worries, and uncertainty about donation. Therefore, the importance of a universal donor recruitment and management program focused on reducing donation-related insecurities through tailored messages and strategies for each ethnic group is clear. According to a report [6], improving the retention rate of Korea’s volunteer donors is a cost-effective method, since 1% improvement has an equivalent effect of recruiting 28,000 new volunteer donors. Maintaining a continued increase in the number of volunteer donors while also strengthening efforts to manage retention rates as a means of increasing actual donation are also important in terms of the efficient use of the national budget [7].

Although the hematopoietic stem cell donation (HSCD) process is generally considered safe, health risks, discomfort, and side effects in donors have been reported. When two HLAs are matched, and the donor chooses to proceed with the donation, they have to make daily visits to the hospital for four days to receive granulocyte-colony stimulating factor (G-CSF) injections for peripheral blood hematopoietic stem cell (PBSC) mobilization prior to hospitalization. HSCs are collected during the hospitalization period.

According to the literature, the procedure of HSCD may cause physical and psychological discomfort to the donor. For example, the procedure may cause several side effects, making the donation an altruistic action rather than simple sharing [3]. Most known side effects of HSCD are physical discomfort such as bone and joint pain, headache, fatigue, fever, insomnia, nausea, dyspnea, edema, and rash [8]. Meanwhile, only a few studies have reported on psychological discomfort, e.g., depression [9] and regret [2]. Therefore, there is a need to examine which types of discomfort are experienced by donors throughout the entire HSC donation process. Moreover, it has been reported that healthcare providers, patients, and family members do not consider donors as people requiring care and, therefore, have less overall interest in donors than in recipients [10]. It must be recognized that establishing an appropriate donor management system based on donor experience is as important as the health procedures for the well-being of the recipient.

According to the literature, female donors are at higher risk than males for apheresis, bone-marrow-related adverse reactions [11,12], and serious complications [12], and are twice as likely to require extended hospitalization [11]. Most donors showed a positive attitude toward HSCD 1 year post-donation [13]. Most of the literature, however, has focused on physical discomfort, overlooking psychological discomfort. This study was conducted to provide basic data for the development of education and intervention programs for future HSC donors by examining the physical and psychological dimensions of HSCD experiences. This study also aims to study the effect of gender on the donors’ discomfort levels.

## 2. Materials and Methods

### 2.1. Research Design and Participants

This study comprises descriptive research. It was approved by a Bioethics Review Committee (DKU202002022). Participants were actual donors registered to C Hematopoietic Stem Cell Bank as potential HSC donors. Research received approval of data collection from the president and the operator of the bank, who also discussed the survey procedure, and posted the research introduction in a periodical of the bank to recruit participants. Among the recruited, participants were chosen by a convenience sample with the following criteria: (1) having donated hematopoietic stem cells in the previous 2 years, (2) agreeing to participate in the study, and (3) having primary school or higher education. Among the participating 138 individuals who donated hematopoietic stem cells from 2017 to 2019, the data of only 131 were used in the analysis, leaving out 7 participants because of incomplete answers. All the participants provided their consent for participation in the study.

### 2.2. Measurements

A literature review was undertaken to identify the types and degrees of physical and psychological discomfort experienced by HSC donors [2,7,14,15]. We also conducted two panel discussions with about 10 past HSCD donors to identify HSCD-related discomfort that the literature did not cover. From a literature review and panel discussions, a comprehensive list of discomfort types was made and a questionnaire was developed by an expert group of three employees of the HSCD registration institute, two professors in nursing, and one physician.

Data were gathered using a self-report questionnaire. The first part consisted of 10 questions regarding the general characteristics of the participants, and 7 questions about their donation experience, such as donation method, motivation, and responses of others. The second part of the questionnaire contained 14 and 11 questions about physical and psychological discomfort, respectively. The degree of physical and psychological discomfort was indicated on a zero (not at all) to ten (very severe) scale. At the end of the questionnaire, we provided an open question about any other discomfort the donor experienced. The provided discomfort types are categorized and analyzed.

### 2.3. Analysis

The collected data were analyzed using the statistical package for social sciences (SPSS Inc., Chicago, IL, USA) version 26.0. Data analysis used descriptive statistics including frequency, ratio, mean, and standard deviation. The differences in physical and psychological discomfort according to gender were analyzed by Mann-Whitney U-test.

### 2.4. Donation Procedure

For easy exposition, we describe the typical process for HSCD as follows. A donation process consists of the following phases: registration phase, decision phase, preparation phase, donation phase, and recovery phase. After the donor registers to the registrar, they are on a waiting list until an HLA match is found (registration phase). Once a match is found, the donor is notified and given 10 days to make a decision (decision phase). If the donor accepts, G-CSF shots are given to the donor for stem-cell mobilization (preparation phase). For donation, the individual is hospitalized for 3 to 4 days for apheresis (donation phase). After donation, the donor recovers (recovery phase).

## 3. Results

### 3.1. General and HSCD-Related Characteristics of the Participants

Among the 131 participants (90 males, 41 females), most were less than 30 years old, and the rest were in their 30s. Socio-demographic characteristics showed that most participants had college education (90.1%) and were not married (74.8%). At the time of donation, participants were employed, students, or in the military services (86.3% in total). Most participants had prior experience with blood donation (87.0%). Forty-two percent of the participants were registered as organ donors and 39.8% had a family history for cancer. Table 1 shows the general characteristics of the participants.

All 131 participants donated their HSCs by the peripheral HSCD method. Their motivation for donation were religious (39.7%), spontaneous decision in a donation campaign (38.9%), contribution to the cure of disease (12.2%), recommendation from others (4.6%), and having an acquaintance with a health problem (4.6%). For 42% of participants, it took 5 years or more from registration to HLA matching; it took less than a year for only 10.7%. Also, 87.8% responded that the information about HSC helped them maintain their will for donation, and 61.1% answered that the medical value of HSCD was the main reason for their donation. During the decision phase, 51.9% experienced disapproval from others. This disapproval was most commonly attributed to misunderstandings about HSCD. (Table 1).

### 3.2. Physical and Psychological Discomfort Experienced During HSCD Process

#### 3.2.1. Frequency and Degree of Discomfort

All 131 participants reported that they experienced physical discomfort during donation (Table 2). The most experienced physical discomforts were Myalgia (72.5%) followed by Bone pain (62.6%), Fatigue (60.3%), and Headache (55.0%). The degrees of physical discomfort (scaled 0 to 10) were in a similar order; the most severe was Myalgia followed by Bone pain, Fatigue, Headache, so on.

Among the participants, 116 (88.5%) responded that they experienced at least one kind of psychological discomfort during HSCD. The most experienced psychological discomfort was Fear (44.3%) and Anxiety (44.3%), followed by Stress (39.7%), Depression (31.3%), and Loneliness (31.3%). The most severe psychological discomforts (scaled 0 to 10) were, in a decreasing order, Fear, Anxiety, Stress, Loneliness, Depression, Regret, Ambivalence, and Others.

#### 3.2.2. Discomfort Period, Duration, and Degree of Disruption to Daily Life

The most severe physical discomfort was experienced in the preparation phase by 44.3% of the participants, followed by the donation (34.3%) and recovery (21.4%) phases. Such physical discomfort lasted for less than one week for 85.5% of participants. The impact of physical discomfort on daily life was endurable for 80.1% of participants, and severe or worse for 19.9% (Table 3).

The most severe psychological discomfort was experienced in the preparation phase by 37.4% of the participants, followed by the donation and decision phases in decreasing order. Most psychological discomfort lasted less than 1 week (91.4%). The impact of psychological discomfort on daily life was endurable for 98.3% of participants (Table 3).

### 3.3. Physical and Psychological Discomfort Experienced During HSCD Process According to Gender

An analysis of physical and psychological discomfort by gender showed statistically significant differences in rash (Z = −2.123, *p* = 0.034), fear (Z = −2.851, *p* = 0.004), and anxiety (Z = −1.861, *p* = 0.044) (higher for female donors) (Table 4).

### 3.4. Other Types of Discomfort Experienced During HSCD Process

The 48 participants (36.6%) responded to the open question asking about other types of discomfort (Table 5). Sixteen participants answered “noncooperative attitude of medical personnel”, 10 felt discomfort due to the “curiosity about the receiver”, 8 mentioned “social environment”, and others mentioned “distance to the hospital”, “family concerns”, etc.

## 4. Discussion

All HSC donors who participated in this study responded that they experienced at least one or more forms of physical discomfort during the HSCD process. The types of physical discomfort experienced by the participants consisted, in decreasing order of frequency, of myalgia, bone pain, fatigue, and headache, corroborating a previous study [8]. Although G-CSF has mild side-effects in general, donors may develop various symptoms leading to physical discomfort which can also cause changes to some test results [16]. The process of collecting HSC also causes pain and side-effects to donors [3]. HSC donors have reported having to bear too much burden on their daily lives before, during, and after their donation [7]. Therefore, healthcare providers must consider the safety of donors as their first priority [14], and efforts should be made to minimize donor discomfort and confusion throughout the donation process.

The phase in which the physical discomfort was most severe was the preparation phase, followed by the donation and recovery phases, consistent with the findings of Lee’s study [10], which suggested that donors experience procedure-related discomfort and pain extending from the HSCD preparation phase to after the donation. Others have argued [7] that the active management of the donors’ well-being after donation is needed, as HSC donors experience difficulties in their daily lives for a certain period of time even after the donation. The present study also found that 35.1% of donors reported disruption to their daily lives due to physical discomfort. Some donors experienced pain up to one year after donation [17]. Healthcare providers should conduct risk assessments of the donors to identify high-risk donors who require special follow-up monitoring or self-management [14]. Among the types of physical discomfort, the degree of rash was greater for female donors with statistical significance. It was reported that female donors have adverse reactions because of pharmacokinetic, immunological, and hormonal differences [18]. Further research on the degree of rash of donors is called for, with a thorough evaluation considering personal characteristics such as body weight.

According to a prior study, some donors were able to see a beneficial side to the process, i.e., saving a life by enduring inconvenience from minor and major discomforts while going through the HSCD preparation phase and the process of actual donation and recovery, and perceived the donation process as a valuable experience in their lives [7]. Conversely, HSCD was also perceived as a negative experience by a qualitative study on HSC donors [19]; donors feel neglected due to medical dismissiveness and family inattention, feel responsible for their recipient’s outcome, and experience profound sadness and disappointment when transplantation fails. In fact, when depression scores were measured six months after donation, donors whose recipients died had significantly higher scores than those whose recipients lived [9], which shows that different situations and outcomes can have different impacts on HSCD experience.

In this study, the phase in which the psychological discomfort was most severe was the preparation phase. As 12.2% of the donors responded that they experienced discomfort during the decision phase, about 50% of donors experienced psychological discomfort prior to the donation phase. These results suggest the necessity of a donor-centered, systematic preparation program for managing donors throughout the entire process.

This study found that female donors showed higher degrees of anxiety than males during HSCD. The anxiety was greatest just before initiating stem cell mobilization in the preparation phase; 33% of donors experienced mild to moderate anxiety and 7% experienced severe anxiety [20]. Although the anxiety levels are not so severe as to directly influence the blood composition, e.g., platelet count [21], the experience of high anxiety for female donors can give rise to negative memories about HSCD, so that they hesitate about future donations and discourage others from undertaking HSCD donation. Therefore, during the preparation and donation phases (before apheresis), anxiety should be identified and addressed, and interventions for mitigating anxiety are necessary. Fear was also higher for female donors. According to Kim, Choi, and Kim [22], the biggest reason for avoiding HSCD was fear without reason (40%). As fear can impose a psychological burden on donors from the registration phase to the donation phase, correct information and emotional support must be provided throughout the HSCD.

A study assessing the general discomfort and degree of restriction to daily life experienced by HSC donors [8] suggested that being female and going through bone marrow and PBSC collection via central line are predictors of general discomfort. One point to note is that being pessimistic about donating a second time or deciding not to do so were included as predictors of general discomfort, indicating that experience of the donation process can affect willingness to donate in future. Therefore, proper orientation on various issues that may arise during the HSCD process is necessary prior to actual donation. Furthermore, it is necessary to help people to develop positive views on HSCD by providing management methods and sufficient education on how to respond when a problem occurs.

The authors of [14] argued for the need of assessing HSC donor psychological states, especially regarding motives. We believe that this is not only a tool that can be used to improve consent rates for donation, but that is also necessary to check the psychological status of donors and help them to be positively motivated. Appropriate education should be provided, as ambivalence on the part of individuals considering donating is a better predictor of a negative donation experience than actual physical difficulties [5]. We believe that it is of the utmost importance to provide opportunities for donors to view their donation process as a positive experience by establishing a system for assessing and managing their psychological state from the decision phase onward.

During the recovery phase after HSCD, donors should be provided with an opportunity to explore their psychological discomfort through a process of sharing their donation experience, and help should be offered to mitigate any psychological discomfort. Also, donor feelings of responsibility and guilt over the outcome of the donation should be managed in order to allow them to regard their action as a positive life experience. To this end, healthcare providers from various fields should develop and implement an intervention program that can thoroughly assess the psychological sate of HSC donors and contribute to improving their psychological well-being; based on the results of such assessments, an efficient management program should be developed. Although most HSC donors carry on with their daily lives without developing complications, there is still a possibility that this can occur. Therefore, all of the involved teams must work in close collaboration to determine the best method for performing HSCT while preserving donor health [23].

In this study, donors responded that the noncooperative attitudes of healthcare providers were a major source of discomfort experienced during the HSCD process, in addition to physical and psychological discomfort. This is also evident in a previous study [7], which cited difficulties experienced by donors, including receiving inadequate information and delays in the process due to the lack of understanding of medical staff. Because healthcare providers are a source of health information and advice, it is necessary to assess their knowledge, attitudes, and behavior regarding HSCD [24]. Therefore, healthcare providers should have appropriate knowledge of and supportive attitudes towards HSCD. The need to include HSCD education in school curricula has been argued for [24]; this could generate more interest in HSC donor registration [25]. In other words, through active education, healthcare providers should be the main agents with sufficient knowledge and positive views of HSCD to help provide respect and support for donors [7].

## 5. Limitation

This study is limited in that it was based on the recollection of the participants about their donation in the past, instead of the data collected during the actual donation procedure. The study was also based on participants who donated through a single HSC bank, so generalizations shouldn’t be made, and repeated research with a larger sample size is called for.

## 6. Conclusions

This study was conducted to pave way for an efficient management program for HSC donors by identifying the forms of physical and psychological discomfort experienced by HSC donors throughout the donation process, i.e., from registration to recovery. The results showed that all donors who participated in the study experienced one or more forms of physical discomfort, while most also experienced psychological discomfort, indicating that establishing a management program for donors is urgently needed.

Such a program should encompass a wide range of aspects, assessing and managing physical, psychological, and social-environmental discomfort that may arise during all phases of the donation process. Healthcare providers should develop a multifaceted intervention program to support and advocate for donors during all phases of HSCD from the donor’s point of view; in this way, the effects of such interventions will be maximized.

In the future, it will be necessary to prospectively investigate the physical and psychological discomfort experienced by donors during each phase of the HSCD process, and to develop intervention programs for each donation phase for donors; their effects should be evaluated through further research.

## Figures and Tables

**Table 1:** General and HSCD (Hematopoietic Stem Cell Donation)-related characteristics of participants (N = 131).

Characteristics	Categories	*n* (%)
Sex	Male	90 (68.7)
	Female	41 (31.3)
Age (years)	20~29	78 (59.5)
	30~39	39 (29.8)
	40~49	11 (8.4)
	50~59	3 (2.3)
Level of education	below high school graduation	13 (9.9)
	above college graduation	118 (90.1)
Marital status	Not married	98 (74.8)
	Married	33 (25.2)
Religion	Yes	69 (52.7)
	No	62 (47.3)
Occupation at the time of donation	Student	32 (24.4)
	Soldier	8 (6.1)
	Employed or self-employed	73 (55.8)
	Other (including homemakers)	18 (13.7)
Living arrangement	alone	47 (35.9)
	with family	77 (58.8)
	with relatives or friends	7 (5.3)
Blood donation experience	Yes	114 (87.0)
	No	17 (13.0)
Registered as an organ donor	Yes	55 (42.0)
	No	76 (58.0)
Family member with cancer or history of cancer	Yes	52 (39.8)
	No	79 (60.2)
Donation method	Bone marrow donation	0 (0.0)
	Peripheral blood stem cell donation	131 (100.0)
Motivating factor for registering	Recommendation from others	6 (4.6)
	Personal or religious beliefs	52 (39.7)
	Medical value	16 (12.2)
	Having an acquaintance who needs HSCD	6 (4.6)
	Spontaneous decision in a donation campaign	51 (38.9)
Duration of HLA (Human Leucocyte Antigens) coincidence	within 1 year	14 (10.7)
	1 to 2 years	35 (26.7)
	3 to 4 years	27 (20.6)
	5 years or more	55 (42.0)
If information about HSC was helpful in maintaining willingness to donate	Helpful	115 (87.8)
	Not helpful	16 (12.2)
Most helpful motive when making your HSCD decision	Medical value of HSCD	80 (61.1)
	Personal stories of patients or experiences of actual donors	39 (29.8)
	Efforts to publicize HSCD promotion	12 (9.1)
Presence of disapproval when you consulted others about donation	Yes	68 (51.9)
	No	63 (48.1)
Disapproval reasons if disapproved (*n* = 68).	Misconception about HSCD (fear and after-effects)	52 (76.5)
	Lack of respectful treatment of donors when undergoing HSCD	4 (5.9)
	Lack of social support in culture of donation (school, work, etc.)	3 (4.4)
	Others’ lack of spirit to be of service to society	9 (13.2)

**Table 2:** Frequencies and degrees of Physical and Psychological discomfort during HSCD (N = 131).

Physical Discomfort			Psychological Discomfort		
	*n* (%)	Mean (SD)		*n* (%)	Mean (SD)
Bone pain	82 (62.6)	3.48 (3.52)	Fear	58 (44.3)	1.61 (2.41)
Headache	72 (55.0)	2.51 (3.22)	Anxiety	58 (44.3)	1.44 (2.29)
Myalgia	95 (72.5)	3.89 (3.40)	Stress	52 (39.7)	1.27 (2.28)
Nausea	57 (43.5)	1.30 (2.33)	Depression	41 (31.3)	0.51 (1.06)
Vomiting	49 (37.4)	0.79 (1.80)	Regret	39 (29.8)	0.46 (1.12)
Diarrhea	47 (35.9)	0.50 (1.04)	Loneliness	41 (41.3)	0.57 (1.31)
Fatigue	79 (60.3)	3.22 (3.59)	Ambivalence	31 (23.7)	0.40 (0.97)
Rash	49 (37.4)	1.19 (2.54)	Others	3 (2.3)	0.10 (0.69)
Insomnia	55 (42.0)	1.43 (2.66)			
Allergic	44 (33.6)	0.56 (1.38)			
Others	13 (9.9)	0.68 (2.27)			

**Table 3:** Discomfort period, duration, and degree of disruption to daily life (N = 131).

Variables	Categories	Physical	Psychological
		*n* (%)	*n* (%)
Period of greatest discomfort	Never	0 (0.0)	15 (11.4)
	Registration phase	0 (0.0)	0 (0.0)
	Decision phase	0 (0.0)	16 (12.2)
	Preparation phase	0 58 (44.3)	49 (37.4)
	Donation phase	0 45 (34.3)	36 (27.5)
	Recovery phase	0 28 (21.4)	15 (11.5)
	Total N	131	131
Duration of the period of greatest discomfort	1~2 days	65 (49.6)	64 (55.2)
	3 days ~ less than 1 week	47 (35.9)	42 (36.2)
	1 week ~ less than 2 weeks	9 (6.9)	6 (5.2)
	2 weeks ~ less than 1 month	10 (7.6)	0 (0.0)
	1 month or longer	0 (0.0)	4 (3.4)
	Total N	131	116
Degree of disruption to daily life due to discomfort	Very severe	4 (3.1)	0 (0.0)
	Severe	22 (16.8)	2 (1.7)
	Moderate but endurable	51 (38.9)	24 (20.7)
	Slightly	24 (18.3)	19 (16.4)
	Not at all	30 (22.9)	71 (61.2)
	Total N	131	116

**Table 4:** Physical and psychological discomfort according to gender (N = 131).

		Male (*n* = 90)		Female (*n* = 41)		Z (*p*)
		*n* (%)	Mean (SD)	*n* (%)	Mean (SD)	
**Physical discomfort**	Bone pain	56 (62.2)	3.68 (3.55)	26 (63.4)	3.03 (3.48)	−0.611 (0.541)
	Headache	47 (52.2)	2.68 (3.30)	25 (61.0)	2.15 (3.08)	−0.562 (0.574)
	Myalgia	67 (74.4)	4.07 (3.53)	28 (68.3)	3.50 (3.11)	−0.831 (0.406)
	Nausea	35 (38.9)	1.21 (2.47)	22 (53.7)	1.50 (2.00)	−1.604 (0.109)
	Vomiting	30 (33.3)	0.80 (1.89)	19 (46.3)	0.76 (1.63)	−0.523 (0.601)
	Diarrhea	29 (32.2)	0.50 (1.16)	18 (43.9)	0.50 (0.70)	−0.748 (0.454)
	Fatigue	51 (56.7)	2.87 (3.55)	26 (63.4)	4.00 (3.63)	−1.148 (0.251)
	Rash	28 (31.1)	0.75 (2.04)	21 (51.2)	2.15 (2.58)	−2.123 (0.034)
	Insomnia	34 (37.8)	1.38 (2.71)	21 (51.2)	1.53 (2.58)	−0.902 (0.367)
	Allergic	27 (30.0)	0.45 (1.36)	19 (46.3)	0.80 (1.41)	−1.317 (0.188)
	Others	7 (7.8)	0.42 (1.78)	6 (14.6)	1.26 (3.06)	−1.227 (0.220)
**Psychological discomfort**	Fear	33 (36.7)	1.01 (1.90)	25 (60.9)	2.92 (2.89)	−2.851 (0.004)
	Anxiety	36 (40.0)	1.05 (1.94)	22 (53.6)	2.30 (2.75)	−1.861 (0.044)
	Stress	30 (33.3)	0.98 (1.97)	22 (53.6)	1.92 (2.77)	−1.864 (0.062)
	Depression	27 (30.0)	0.47 (1.01)	15 (36.6)	0.61 (1.16)	−0.549 (0.583)
	Regret	24 (26.7)	0.26 (0.44)	16 (39.0)	0.92 (1.85)	−1.470 (0.142)
	Loneliness	27 (30.0)	0.59 (1.42)	14 (34.1)	0.53 (1.06)	−0.398 (0.691)
	Ambivalence	24 (26.7)	0.38 (0.94)	11 (26.8)	0.46 (1.06)	−0.147 (0.883)
	Others	3 (3.3)	0.15 (0.84)	0 (0.0)	0.00 (0.00)	−0.961 (0.337)

**Table 5:** Other discomfort during HSCD process (N = 48).

Open Questions	**N (%)**
Noncooperative attitude of medical personnel (insufficient explanation, attitude toward donor, etc.)	16 (33.3)
Problems related to distance to the hospital (distance to the hospital when visiting during donation preparation period and distance to the medical facility where hospitalization is available)	4 (8.3)
Social environment (taking vacation days, uncomfortable gaze, etc.)	8 (16.7)
Family concerns	4 (8.3)
Curiosity about the receiver	10 (20.8)
Others	6 (12.5)

## References

[1] Gratwohl A., Baldomero H. (2009). Trends of hematopoietic stem cell transplantation in the third millennium. Curr. Opin. Hematol..

[2] Park M.P., An S.H. (2017). Study on the activation plan for improving hematopoeitic stem cells donation. Bioeth. Policy.

[3] Lyu J.Y. (2016). Unrelated Hematopotopoietic Stem Cell Donor Experiences. Master’s Thesis.

[4] Ministry of Health & Welfare (2018). 2018 Guidelines of Hematopoietic Poietic Stem Cell Donation.

[5] Switzer G.E., Bruce J.R., Myskovsky L., DiMartini A., Shellmer D., Confer D.L., Abress L.K., King R.J., Harnaha A.G., Ohngemach S. (2015). Race and ethnicity in decisions about unrelated hematopoietic stem cell donation. Blood.

[6] Kwon S.Y., Lee M.J., Lee J.H., Cho N.S. (2016). Unrelated hematopoietic stem cell donor recruitment of the Korean Red Cross: 20 years experience. Korean J. Blood Transfus..

[7] Yoo S.Y., Kim M.O., Kim T.G., Beom S.H. (2019). Experiences of unrelated hematopoietic stem-cell donors and experts of relevant institutions. Korean J. Adult Nurs..

[8] Lee M.H., Jang J.H., Min H.J., Jang H.I., Nah J.H., Lyu C.J., Han K.S., Won J.H., Lee Y.H., Chong S.Y. (2017). Predictors of general discomfort, limitations in activities of daily living and intention of a second donation in unrelated hematopoietic stem cell donation. Bone Marrow Transpl..

[9] Chang G., McGarigle C., Koby D., Antin J.H. (2003). Symptoms of pain and depression in related marrow donors: Changes after transplant. Psychosomatics.

[10] Lee Y.J. (2010). Experiences of the Allogeneic Hematopoietic Stem Cell Transplantation Donors. Master’s Thesis.

[11] Pulsipher M.A., Chitphakdithai P., Logan B.R., Shaw B.E., Wingard J.R., Lazarus H.M., Waller E.K., Seftel M., Stroncek D.F., Lopaz A.M. (2013). Acute toxicities of unrelated bone marrow versus peripheral blood stem cell donation: Results of a prospective trial from the National Marrow Donor Program. Blood.

[12] Yuan S., Ziman A., Smeltzer B., Lu Q., Goldfinger D. (2010). Moderate and severe adverse events associated with apheresis donations: Incidences and risk factors. Transfusion.

[13] Butterworth V.A., Simmons R.G., Bartsch G., Randall B., Schimmel M., Stroncek D.F. (1993). Psychosocial effects of unrelated bone marrow donation: Experiences of the National Marrow Donor Program. Blood.

[14] Billen A., Madrigal J.A., Shaw B.E. (2014). A review of the hematopoietic stem cell donation experience: Is there room for improvement?. Bone Marrow Transpl..

[15] Beom S.H., Kim E.J., Kim M., Kim T.G. (2016). Unrelated hematopoietic stem cell registry and the role of the hematopoietic stem cell bank. Blood Res..

[16] Lee Y.H. (2005). The Measures for Improving Database Management of the Bone Marrow Donors.

[17] Pulsipher M.A., Logan B.R., Kiefer D.M., Chitphakdithai P., Riches M.L., Rizzo J.D., Anderlini P., Leitman O.F., Kobusingye H., Besser R.M. (2019). Related peripheral blood stem cell donors experience more severe symptoms and less complete recovery at one year compared to unrelated donors. Haematologica.

[18] Rademaker M. (2001). Do women have more adverse drug reactions?. Am. J. Clin. Dermatol..

[19] Gracia M.C., Chapman J.R., Shaw P.J., Gottlieb D.J., Ralph A., Craig J.C., Tong A. (2013). Motivations, experiences, and perspective of bone marrow and peripheral blood stem cell donors: Thematic synthesis of qualitative studies. Biol. Blood Marrow Transpl..

[20] Gutieerrez-Aguirre C.H., Rodrigyez G.C., Garza-Salazar F.D.L., Riojas R.S., Leoh A.G.D., Pedraza P.R.C., Guerrero-Gonzalez G., Jaime J.C., Ruiz-Arguelles G.J., Gomez-Almaguer D. (2019). Moral distress, anxiety, and others physical symptoms related to hematopoietic stem cell donation. Blood.

[21] Yilmaz S., Eker İ, Elçi E., Pekel A., Çetinkaya R.A., Ünlü A., Açıkel C., Avcı I.Y. (2019). Evaluating the effect of donor anxiety levels and lifestyle characteristics on the activation of platelet concentrates. Blood Res..

[22] Kim K.C., Choi W.J., Kim T.K. (2019). The 2019 hematopoietic stem cell and cord blood (CB) donation public awareness survey. Health Dise..

[23] Hequet O., Mialou V., Audat F., Wattel E., Chapel V., Revesz D., Jouet J.P., Fisseaux B., Saoud M., Michallet M. (2016). Management of psychiatric complications in unrelated donor before unrelated peripheral hematopoietic stem cell collections. J. Blood Med..

[24] Narayanan P., Wolanskyj A., Ehlers S.L., Litzow M.R., Patnaik M.S., Hogan W.J., Hashmi S.K. (2016). Medical students’ knowledge, familiarity, and attitudes towards hematopoietic stem cell donation. Biol. Blood Marrow Transpl..

[25] Sikora A., Wiórkowski K., Szara P., Drabko K. (2014). Knowledge and attitude of Lublin universities students’ toward the opportunity of becoming unrelated bone marrow donor. Folia Med. Cracov..

